# Rating scale measures are associated with Noldus EthoVision-XT video tracking of behaviors of children on the autism spectrum

**DOI:** 10.1186/2040-2392-5-15

**Published:** 2014-02-18

**Authors:** Ira L Cohen, Judith M Gardner, Bernard Z Karmel, Soh-Yule Kim

**Affiliations:** 1 Department of Psychology, New York State Institute for Basic Research in Developmental Disabilities, 1050 Forest Hill Road, Staten Island, NY 10314, USA; 2 Department of Infant Development, New York State Institute for Basic Research in Developmental Disabilities, 1050 Forest Hill Road, Staten Island, NY 10314, USA

**Keywords:** Autism spectrum disorders, Automated tracking, Behavioral assessment, Rating scales, Thigmotaxis, Treatment measures

## Abstract

**Background:**

Children with Autism Spectrum Disorder (ASD) show unusual social behaviors and repetitive behaviors. Some of these behaviors, e.g., time spent in an area or turning rate/direction, can be automatically tracked. Automated tracking has several advantages over subjective ratings including reliability, amount of information provided, and consistency across laboratories, and is potentially of importance for diagnosis, animal models and objective assessment of treatment efficacy. However, its validity for ASD has not been examined. In this exploratory study, we examined associations between rating scale data with automated tracking of children’s movements using the Noldus EthoVision XT system; i.e., tracking not involving a human observer. Based on our observations and previous research, we predicted that time spent in the periphery of the room would be associated with autism severity and that rate and direction of turning would be associated with stereotypies.

**Methods:**

Children with and without ASD were observed in a free-play situation for 3 min before and 3 min after Autism Diagnostic Observation Scale – Generic (ADOS-G) testing. The Noldus system provided measures of the rate and direction of turning, latency to approach and time spend near the periphery or the parent.

**Results:**

Ratings of the severity of maladaptive social behaviors, stereotypies, autism severity, and arousal problems were positively correlated with increases in percent time spent in the periphery in the total sample and in the ASD subset. Adaptive social communication skills decreased with increases in the percentage of time spent in the periphery and increases in the latency to approach the parent in the ASD group. The rate and direction of turning was linked with stereotypies only in the group without ASD (the faster the rate of a turn to the left, the worse the rating). In the ASD group, there was a shift from a neutral turning bias prior to the ADOS assessment to a strong left turn bias after the ADOS assessment. In the entire sample, this left turn bias was associated with measures of autism severity.

**Conclusion:**

Results suggest that automated tracking yields valid and unbiased information for assessing children with autism. Turning bias is an interesting and unexplored measure related to autism.

## Background

Autism spectrum disorders (ASD) are characterized by atypical socialization and communication along with repetitive and ritualistic behaviors and problems with arousal regulation. Such behaviors are often quantified by rating scales or by more objective measures such as coding of behaviors from video samples. While valuable, such measures require human judgment which can be affected by a number of factors including understanding of the items, educational level of the informant, cultural background of the child or informant, and informant expectancies which, in turn, can contribute to placebo effects. One way of countering these effects is through the use of automated systems often used in animal studies for detecting responses and which are starting to be used for humans.

Automated devices to detect stereotypic behaviors, for example, have been shown to be a promising alternative to rating scales, as this minimizes the role of human decision making and can provide much more quantitative and dynamic information [1,2]. Further, eye tracking devices have yielded important information relevant to both early detection [3] and toward understanding the nature of the social deficits in autism [4]. Automated detection of social interactions in this cohort has, however, not been developed although it has been explored in animal models of ASD. For example, automated detection of social interaction and social preference has been developed for mice in the hope of mimicking the social deficits seen in autism. One such task involves measuring the percentage of time spent with an unfamiliar mouse relative to a conspecific as a measure of social preference [5,6].

We have clinically observed that children with ASD, given free choice to move in our observation room, often stay away from the parent and remain near the periphery both sitting and exploring the toys and books that are available, or moving around the periphery watching themselves in our one-way mirror and/or touching the walls. Similar movement patterns have been observed by us in toddlers at risk for ASD. As a result, the amount of time spent near the parent is relatively small relative to the amount of time spent in the periphery. As with the animal tasks, such propensities can also be automatically quantified using commercially available systems.

The Noldus EthoVision-XT system is one such system that has been utilized to track movements of animals in laboratory environments [7]. Using an overhead camera and frame grabber, the software can track animals based on their black or white shading or by color marking. Such tracking has advantages over subjective measures in terms of reliability and amount of information provided and consistency across laboratories, and is potentially of importance for assisting with diagnosis, providing measures that may be less susceptible to cultural influences, and in providing objective measures of treatment efficacy which may be less susceptible to observer bias. In an unpublished study, the EthoVision-XT system has been explored as a means of tracking people in a human-sized version of the Morris water maze (http://www.noldus.com/documentation/human-spatial-orientation-and-way-finding-analysis-ethovision-real-arena-maze).

In this exploratory study, we examined the validity of automated tracking for children with ASD by examining associations between the obtained measures with autism-relevant rating scale data obtained from a parent or a clinician. Measurements were taken in a free play situation before and after diagnostic evaluations with the Autism Diagnostic Observation Scale-Generic (ADOS-G) [8] and consisted of quantifying the amount of time spent near the parent or near the periphery, as well as the average speed and direction of turning of the child’s body during the observation. The latter was of interest because of an automated study of stereotyped spinning behavior in people with ASD, which indicated that such spinning had a left turn bias [1], and because of the often reported observations of atypical lateralization in ASD.

We hypothesized that these tracking data would be associated with a variety of measures indicative of the severity of ASD with the amount of time spent in the periphery showing the strongest effect. We also hypothesized that a left-turn bias would be associated with stereotyped behaviors.

## Methods

### Participants

The participants were 36 out of 40 children consecutively referred for diagnosis or follow-up evaluations of ASD. The four cases not included were one child, 22 months of age, whose diagnosis was unclear; one child whose primary language was not English (precluding an ADOS assessment); one child whose parent sat in the wrong part of the room; and one who failed to remain after the ADOS-G assessment. The mean ± SD age of these 36 cases was 5.8 ± 3.1 years. Males composed 83% of the sample. Four cases were seen again between 7 and 12 months later (3 males, 1 female) and their data were also included in the analyses, thus yielding 40 data points.

Twenty-seven children were diagnosed as being on the autism spectrum based on DSM-IV-TR criteria [9]: autistic disorder (n = 16) and pervasive developmental disorder (PDD)–not otherwise specified (n = 11). The remaining nine children had diagnoses of attention-deficit hyperactivity disorder (n = 4), anxiety disorder–not otherwise specified (n = 3), mixed receptive-expressive language disorder (n = 1), and global developmental delay (n = 1).

### Observation room and EthoVision-XT 8.0 system

Children were evaluated in a large room, 3.18 m in length, 4.85 m in width, and 2.44 m in height. A color CCD camera (Polestar II Everfocus) with a wide angle lens was mounted in the center of the ceiling with the bottom of the lens located 30 cm from the ceiling. The signal from the camera was processed by a Euresys™ Picolo U4H.264 frame grabber and encoder board housed in a Dell™ Precision Desktop computer.

**Figure 1:**
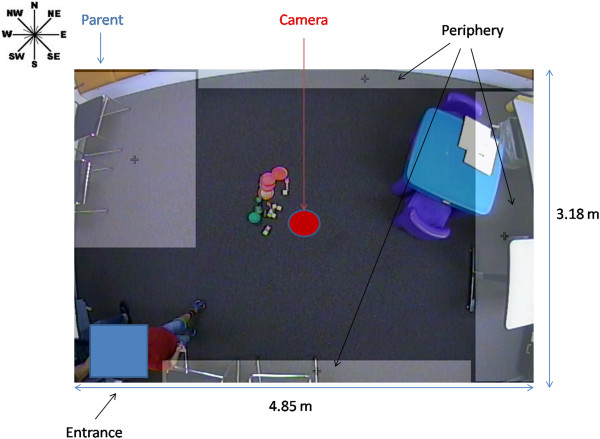
**Top down view of observation room.** Camera is mounted in the center of the ceiling. Shaded regions of interest (ROIs) are parent (top left), and combined one-way mirror (top), storage (right), and front (bottom) area of the arena (+ denotes ROI center).

EthoVision-XT 8.0 software tracked the location of the child by the color of the shirt he/she was wearing (color marker tracking at a rate of 29.97 samples/sec), providing x,y coordinates relative to the center of the room that were later processed off-line. In our setup, a red shirt provided the best contrast against the background. In cases where the child did not wear a red shirt, the parent was asked to place red vinyl tape (3M™ #471 – 3 in width) on the shoulders and upper arms of the child’s shirt. The tape could easily be removed without harming the shirt. The EthoVision system computed the area of the red target and then used the center of this area to define the location of the subject.Figure 1 shows a top-down view of the area of the observation room that was coded, i.e., the “arena” (note some “fish-eye” distortion in the photo). It shows the entrance, location of the camera (center of the arena), testing table, storage areas, and location of chairs. The north side of the room has a one-way mirror with storage cabinets underneath it. The east side of the room had two tables where the ADOS-G materials were kept. The light gray rectangles show two basic regions of interest (ROIs) in the arena; i) The ROI surrounding the parent (marked by the two chairs where the parent sat (and child as well if he/she so chose to do so) and ii) the ROI marking the periphery of the room away from the parent. Movement into any of these areas was considered as being within the periphery. The “+” signs indicate the centers of the ROIs.

### Tracking protocol

The room was set up as shown in Figure 1. The protocol was modified from that developed by Gardner [10] to study social behavior and arousal in toddlers in an open field situation. Toys were placed on the floor and table top. The parent and child were introduced to the observation room and the parent was instructed to sit in the northwest corner and asked to complete the Aberrant Behavior Checklist-Community version (ABC-C) [11]. The parent was told the child could play with the toys and was free to roam around the room. The examiner then left the room for 3 min (measured with an electronic timer). After this period, the examiner returned and tested the child with the ADOS-G while the parent remained to watch. The mean ± SD time to administer the ADOS was 24 ± 9.7 min and varied with the module used. After the ADOS-G testing was done, the examiner cleaned up the materials, set up the room as before, asked the parent to complete the ABC-C and then the examiner exited the room. The child was then tracked for an additional 3 min of free play with the parent present. Tracking data were gathered for both 3 min periods and examined for differences across the two time periods. All but three parents remained focused on filling out the ABC-C. Two children without ASD and one child with ASD approached their parent during the final 3 min period requiring the parents to respond to their child’s bid for attention.

### Data filtering

Filtering of the data prior to computation of the predictor measures was necessary for two reasons. First, there were instances where the system mistook another red object as the subject (e.g., red shoes or toys). These frames were manually deleted and then substituted by linear interpolation from the closest non-missing frames. Second, as the subject moved, wobbling was noted from one frame to the next (e.g., the center would move from one shoulder to the other). The wobbling was minimized in two different ways. A smoothing algorithm included in the EthoVision package that used a two-degree locally weighted scatterplot smoothing function (“lowess” function) was applied to 10 frames before and after the center point [12]. Frames closest to the center exerted the greatest influence. After this lowess smoothing, an additional minimum distance movement of ≥2.54 cm criterion between frames was applied to the entire data log for each child in order to further eliminate wobbling, thus minimizing effects of small body movements.

### Predictor variables from tracking data

Since ASD is a disorder of social communication and repetitive behavior, we focused on measures most relevant to these symptoms based on the ROIs defined above:

i. Parent directed: The focus here was on measures related to the parent’s location in the room. These included percentage of session time spent in parent ROI and latency(s) to approach the parent ROI (measured from the time the ADOS examiner walked out the door).

ii. Periphery directed: As noted above, we have observed that children with ASD tend to prefer remaining close to the periphery of a room. This thigmotaxis-like behavior may reflect anxiety but, in our setup, could also indicate preference for exploring the toys, watching oneself in the one-way mirror, and/or increasing the space available to engage in repetitive motoric behavior. Measures here included: percentage of session time spent in the periphery ROI closest to child and latency(s) to approach the periphery ROI closest to the child (measured from the time the ADOS examiner walked out the door).

iii. Turning Bias: The speed and direction of motion taken by the child when he/she was in the room was examined as a proxy measure of repetitive behaviors as noted above. Our measure for this bias was relative angular velocity (RAV).

RAV is the signed change in direction of movement of a subject from one sample to the next per unit time (degrees/sec; ^o^/sec). A clockwise (right) turn, relative to a horizontal line at the center of the room, is scored as a negative value. A counterclockwise (left) turn is scored with a positive value. RAV serves as a measure of the tendency of the subject to turn in one particular direction such as would be found in circling or in choosing to explore objects based on their relative position with respect to where the child was sitting or standing (i.e., to his/her left or right side). Calculation details can be found in the EthoVision-XT 8 manual. As noted above, problems with laterality dominance have been described in the autism literature, especially in those with more severe communication impairment [13,14]. Therefore, the tendency to move in a particular direction was of interest. As a result of the minimum distance moved criterion, RAV was computed only for those movements that exceeded 2.54 cm between two consecutive samples, again to minimize the influence of small body movements.

In order to verify the accuracy of the minimal distance filtering on RAV, a research assistant was asked to wear a red shirt and to go into the observation room and move in small and large circles; first in a counter-clockwise direction and next in a clockwise direction. She executed 20 counter-clockwise and 19 clockwise turns in 125 sec and 111 sec, respectively. The RAV for the counter-clockwise circles was calculated as 59.8°/sec and for the clockwise circles it was calculated as −62.3°/sec; resulting in an estimated 21 counter-clockwise and 19 clockwise 360° circles, respectively.

The accuracy of all measures was also validated by observation of the video of the child’s location in the room and where the system located him or her using the “Integrated Visualization” module which showed graphs of all measures over time along with a concurrent display of the overhead camera view. Thus, using this we could verify that the system was correctly identifying the child as being in a given ROI or moving in a given direction.

These measures were computed for the first and second 3-min periods. The latency and percent time measures did not significantly differ across time periods but RAV did (t (38) = −2.0, *P* = 0.05), as shown in Table 1. Therefore, in all of the correlation tables below, the latency and percentage time measures reflect the average of the first and second 3-min intervals while the RAV measure is shown separately for these two time periods along with the overall mean.

**Table 1:** Pre-post means for tracking measures and pre-post correlations (r); df = 39

	**Mean ± SD Pre**	**Mean ± SD Post**	**t**	***P***	**r**	***P***
Parent Latency (sec)	37.2 ± 52.7	54.2 ± 71.5	−1.3	0.20	0.15	0.36
Percent Time in Parent ROI	30.0 ± 20.3	24.5 ± 26.2	1.2	0.23	0.48	0.00
Periphery Latency (sec)	58.1 ± 72.0	55.0 ± 65.1	0.2	0.84	0.05	0.77
Percent Time in Periphery ROI	24.2 ± 27.4	25.0 ± 25.4	−0.2	0.83	0.61	0.00
Turning Bias RAV (°/sec)	−5.3 ± 26.2	5.6 ± 23.3	−2.0	0.05	0.06	0.73

Also shown in Table 1 is the correlation between these measures from the first to the second 3-min period. Only the percent times spent near the parent or near the periphery were relatively stable across the two time periods. The latency measures were not stable, likely, in part, because of the fact that during the last 3 min, most of the children were not approaching their parents from the same location as in the first 3 min. RAV also differed across time periods as noted above.

Table 2 shows descriptive statistics for the tracking measures. Most had minimal skew and did not differ from a normal distribution. RAV was not normally distributed overall and was negatively skewed and highly peaked in the first 3-min period.

**Table 2:** Descriptive statistics for tracking measures

	**Mean**	**Median**	**SD**	**Skew**	**Kurtosis**	K-S*
Parent Latency (sec)	45.7	31.9	47.4	1.3	1.4	0.2
Percent Time in Parent ROI	27.4	21.7	24.2	1.3	1.4	0.2
Periphery Latency (sec)	56.5	47.9	49.7	0.8	0.2	0.1
Percent Time in Periphery ROI	24.4	19.5	23.7	1.7	2.9	0.2
Turning Bias						
RAV1 (°/sec)	−5.3	−0.6	26.2	−2.6	9.0	***0.3***
RAV2 (°/sec)	5.6	3.2	23.3	0.0	1.4	**0.2**
RAV Mean (°/sec)	0.1	1.9	18.0	−1.4	3.3	0.2

*Kolmogorov-Smirnov test for normality; bold font, *P* <0.05; bold and italics, *P* <0.01.

### Rating scales

#### PDD Behavior Inventory (PDDBI)

Prior to the visit, the parent completed the PDD Behavior Inventory (PDDBI), an informant based tool standardized on children with ASD between 2 and 12 years of age [15-17]. The PDDBI is constructed, *a priori*, in a hierarchical manner. At the first level, the PDDBI is divided into two orthogonal behavioral dimensions: i) Approach-Withdrawal Problems, assessing maladaptive behaviors (higher scores indicate increased severity); and ii) Receptive/Expressive Social Communication Abilities, assessing social communicative competence (higher scores reflect increased competence). Each of these dimensions is comprised of a number of separate behavioral domains best reflecting that dimension.

The PDDBI generates age-normed T-scores (mean (SD) = 50 (10)) for each domain and for each composite score (representing a summary of the domain scores) for children between 1.5 and 12.5 years of age. An Autism Composite score is generated based on those domain T-scores most relevant to a diagnosis of autism. These domain and composite T-scores are normally distributed within the reference sample, enabling complex statistical models to be utilized. While originally developed to measure response to intervention, several of the scores generated from the PDDBI agree very well with diagnoses made by both Autism Diagnostic Interview-Revised and ADOS-G criteria [18]. Table 3 shows the domains of the parent version used in the present study.

**Table 3:** Brief description of the PDD behavior inventory (PDDBI) domains used

**Abbreviation**	**Description and characteristics**
AWP	Approach-Withdrawal Problems Dimension – higher domain T-scores indicate greater severity.
SENSORY	Sensory/Perceptual Approach Behaviors – staring at objects, pica, repetitive toy play, hand flapping, etc.
RITUAL	Ritualisms/Resistance to Change – carrying out rituals or indicating dissatisfaction with a change in the environment or routine.
AROUSE	Arousal Regulation Problems – emotional constriction, hyperactivity, sleeping problems, etc.
FEARS	Specific Fears – fears and anxieties associated with withdrawal from social or asocial stimuli.
AGG	Aggressiveness – aggressiveness toward self or others and associated negative mood states.
SOCIAL DISCREPANCY	A measure of inappropriate social interaction (problems reacting to the approaches of others, understanding social conventions, or initiating social interactions) that corrects for non-vocal social communication skills level (the SOCAPP domain described below).
SEMANTIC/PRAGMATIC DISCREPANCY	A measure of problems with the child’s semantic/pragmatic understanding (aberrant vocal quality, echolalia, and perseveration) that corrects for expressive language abilities (the EXPRESS domain).
REXSAC	Receptive/Expressive Social Communication Abilities Dimension – higher domain scores indicate increasing levels of competence.
SOCAPP	Social Approach Behaviors – non-vocal social communication skills such as paying attention, joint attention, effective use of gesture, imaginative skills, social play skills, imitation skills, etc.
EXPRESS	Expressive Language – ability to speak sounds associated with the English language as well as competence with grammar, tone of voice, and conversational pragmatics.
LMRL	Learning, Memory, and Receptive Language – memory for locations and movement sequences, understanding possessives, prepositions, adverbs, etc.
AUTISM	Autism Composite – a measure of lack of appropriate social communication skills along with repetitive/ritualistic behaviors.

#### Vineland Adaptive Behavior Scales, Second Edition (VABS-II)

Prior to videotaping, the parent was interviewed with the VABS-II [19] to provide an assessment of adaptive abilities and serve as a complement to the PDDBI Receptive/Expressive Social Communication Abilities dimension data.

#### Aberrant Behavior Checklist-Community Version (ABC-C)

As noted above, the parent also completed the ABC-C while the child was tracked during the observation to provide an additional measure of maladaptive behavior besides the Approach-Withdrawal Problems dimension of the PDDBI. For present purposes, we used the ABC-C factor scores developed for people with Fragile X syndrome to characterize these behaviors [20] because of the strong association between ASD and Fragile X and because of the limited information on the factor structure of the ABC-C for people with ASD at the time these data were analyzed.

#### ADOS-G

Finally, the ADOS-G Social Affect, Restricted and Repetitive Behaviors, and Comparison Score were computed.

Raters were blind to the results of the tracking. Data gathered were anonymized prior to analysis. This project was approved by the Institutional Review Board of the New York State Institute for Basic Research in Developmental Disabilities and an informed consent waiver was granted.

### Data analyses

All of the data (including the four repeat data points) were used in order to increase power. Group differences across tracking measures were analyzed using *t*-tests or Mann–Whitney U-tests where appropriate. Pearson correlations were examined between the tracking measures and the rating scale data for the entire sample as well as for the ASD and Not-ASD groups separately (Spearman rho was also examined for the RAV variable but results were quite similar to the Pearson analyses and so are not described herein). The focus here was on both generality and specificity, i.e., we were interested in which of the tracking measures was associated with various classes of behavior, irrespective of the type of rating scale or informant, and which, if any, were specific to measures linked to ASD. The various rating instruments were completed in different situations, at different times, and, in case of the ADOS-G, by different informants. Accordingly, the tables below were grouped by the behavior classes that are common across the different measuring systems in order to examine generality across instruments. A *P* value of ≤0.05 was set and *P* values are shown in all tables. Correction for multiple comparisons was not made as it would be overly strict – this was an exploratory study and the measures within each behavioral class were correlated with one another.

## Results

### Group differences in tracking data

As shown in Table 4, the ASD group showed a greater latency to approach their parents and spent a significantly greater time in the periphery, as predicted. The time spent with the parent, or latency to approach the periphery did not differ across groups. The ASD children were also the ones to show a shift in RAV across the pre-post-time periods while the Not-ASD group showed no such trend (evident in both the *t*- and U-tests).

**Table 4:** **Mean ± SD tracking measures by group and *t*-test (separate variance estimates; df = 38)**

	**ASD**	**Not-ASD**	***t***	***P***
Parent Latency (sec)	53.8 (9.2)	21.5 (10.1)	2.5	0.02
Percent Time in Parent ROI	26.2 (4.5)	31.1 (8.2)	−0.5	0.59
Periphery Latency (sec)	55.1 (10.1)	60.9 (11.4)	−0.4	0.70
Percent Time in Periphery ROI	27.6 (4.8)	14.6 (4.2)	2.1	0.04
Turning Bias*				
RAV1 (°/sec)	−2.8 (3.2)	−13.0 (14.6)	0.7	0.49
RAV2 (°/sec)	12.5 (3.6)	−15.3 (7.3)	3.6	0.00
RAV Mean (°/sec)	4.9 (2.2)	−14.1 (8.4)	2.3	0.04

*Mann–Whitney: U = 144, *P* = 0.86 for RAV1; U = 44, *P* = 0.001 for RAV2; and U = 80, *P* = 0.03 for RAV Mean.

**Figure 2:**
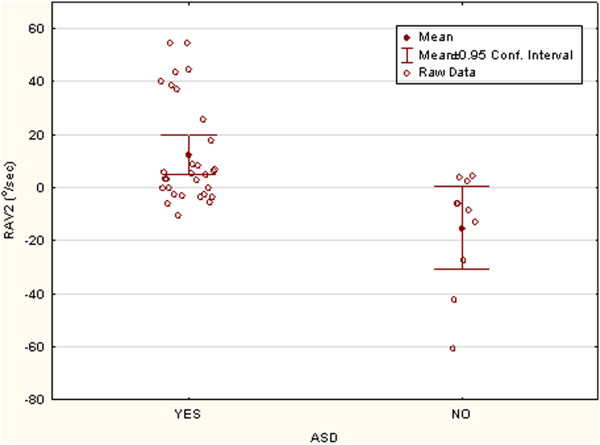
**This figure shows the means, 95% confidence intervals, and raw data for the rate of turning to the left (positive sign) or right (negative sign) in the ASD and Not-ASD groups during the 3-min interval after the ADOS assessment was finished.** Note that the absolute velocity was similar for the two groups but that they markedly differed in turn bias with the ASD group showing a left turn bias.

Figure 2 shows a plot of the group means, 95% confidence interval, and raw data differences on RAV2. Group differences were strong with little overlap. Omitting the repeat data had no effect on these results (t (34) = 3.15, *P* <0.004). Children with ASD had a left turn bias tendency in the angular velocity of their spontaneous motion while the Not-ASD group tended to have a right turn bias. The overall angular velocity was similar in both groups at (mean ± SE) 12.5 ± 19.6°/sec in the ASD group and 15.3 ± 21.8°/sec in the Not-ASD group. Thus, our data indicate that group differences in RAV are vectorial, not scalar, in the sense that small displacements in motion, either by ambulating or sitting and turning, are similar across groups but differ in which direction the displacement occurred.

### Correlational analyses

For all of the analyses below, removing the four repeat data points had no significant effect on the size or direction of the correlations.

### Tracking data and maladaptive social communication

Table 5 shows the correlations between the tracking variables (grouped into Parent, Periphery, and Turning Bias) and the rating scale measures of maladaptive social communication for the entire sample and separately for the ASD and Not-ASD groups. Overall, the tracking measures specifically related to the location of the parent in the room showed little in the way of significant association with almost all rating scale measures of maladaptive socialization, the one exception being the link between ADOS-G Social Affect and latency to approach the parent, an effect driven by the ASD group.

**Table 5:** Correlations between tracking measures and maladaptive social communication severity for all data: All (n = 40), ASD (n = 30), and Not-ASD (n = 10) subsets

	**Parent**		**Periphery**		**Turning bias**		
**Maladaptive Social**							
	**LAT**	**DUR%**	**LAT**	**DUR%**	**RAV1**	**RAV2**	**RAV MEAN**
PDDBI Social Discrepancy							
All	0.19	0.14	−0.11	0.33	0.35	0.21	0.39
*P*	0.096	0.405	0.502	0.039	0.025	0.193	0.012
ASD	0.08	0.19	−0.08	0.40	0.03	0.08	0.09
*P*	0.676	0.310	0.656	0.029	0.882	0.660	0.640
Not-ASD	0.55	0.03	−0.20	−0.19	0.76	0.34	0.80
*P*	0.103	0.927	0.581	0.607	0.011	0.330	0.005
ADOS-G Social Affect							
All	0.46	−0.13	0.07	0.36	0.10	0.53	0.42
*P*	0.003	0.420	0.677	0.023	0.560	0.000	0.008
ASD	0.45	−0.13	0.22	0.28	−0.03	0.26	0.19
*P*	0.013	0.488	0.240	0.135	0.877	0.170	0.316
Not-ASD	−0.52	0.12	−0.20	0.53	−0.48	−0.02	−0.42
*P*	0.120	0.739	.588	0.112	0.164	0.951	0.225
ABC/C Social Avoidance							
All	0.24	−0.03	−0.27	0.32	0.36	0.11	0.33
*P*	0.135	0.862	0.095	0.042	0.024	0.484	0.036
ASD	0.22	−0.05	−0.31	0.39	0.37	−0.09	0.20
*P*	0.240	0.805	0.094	0.033	0.045	0.654	0.299
Not-ASD	0.08	0.10	−0.06	−0.28	0.36	0.29	0.44
*P*	0.826	0.784	0.880	0.441	0.300	0.419	0.204
ABC/C Social Unresponsiveness							
All	0.12	0.06	−0.30	0.25	0.35	−0.05	0.23
*P*	0.477	0.705	0.057	0.125	0.025	0.768	0.162
ASD	0.19	0.07	−0.41	0.44	0.38	−0.10	0.20
*P*	0.317	0.731	0.026	0.015	0.039	0.607	0.308
Not-ASD	0.18	0.01	−0.06	−0.24	0.44	0.36	0.54
*P*	0.627	0.983	0.864	0.510	0.198	0.313	0.109
**Maladaptive Language**							
	**LAT**	**DUR%**	**LAT**	**DUR%**	**RAV1**	**RAV2**	**RAV MEAN**
PDDBI Semantic/Pragmatic Discrepancy							
All	0.27	0.04	−0.25	0.16	0.43	0.29	0.49
*P*	0.096	0.798	0.127	0.324	0.006	0.074	0.001
ASD	0.32	−0.09	−0.28	0.30	0.11	0.17	0.22
*P*	0.089	0.628	0.132	0.112	0.556	0.373	0.245
Not-ASD	0.16	0.30	−0.26	−0.30	0.60	0.38	0.68
*P*	0.668	0.407	0.461	0.404	0.068	0.279	0.031
ABC/C Inappropriate Speech							
All	0.10	0.04	0.20	−0.26	0.18	0.16	0.24
*P*	0.539	0.788	0.226	0.108	0.263	0.321	0.143
ASD	0.18	0.07	0.32	−0.32	0.08	0.45	0.43
*P*	0.345	0.721	0.086	0.088	0.681	0.012	0.018
Not-ASD	0.50	−0.10	−0.17	0.24	0.43	0.43	0.55
*P*	0.141	0.786	0.642	0.513	0.216	0.217	0.096

LAT = Latency (sec); DUR% = Percentage of session time; RAV = Relative angular velocity (°/sec).

**Figure 3:**
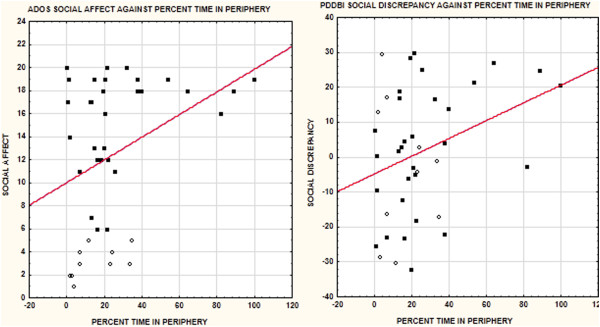
**This figure shows the relation between the Social Affect score on the ADOS (left) and the Social Discrepancy score on the PDDBI (right) against the percentage of time spent in the periphery of the observation room.** ASD cases are in filled circles and Not-ASD cases in open circles. The regression function is for the entire sample. Note that the percentage of time spent in the periphery has more variation in the ASD group than in the Not-ASD group accounting for the stronger correlation for the ASD group in Table 5. Both dependent measures show ceiling effects when the percentage of time spent in the periphery exceeds 50%.

Measures of the tendency to remain in the periphery of the room were, however, associated more broadly with all of the ratings of maladaptive social behaviors with the percent duration spent in the periphery ROI showing the most cross-scale consistency, this effect was again driven by the ASD group. The more time children spent in the periphery, the worse their scores. This measure was not significantly associated, however, with measures of maladaptive language (e.g., echolalia, perseveration, etc.).Figure 3 shows the relation between overall percent time in the periphery against the PDDBI Social Discrepancy and the ADOS Social Affect measures. The ASD group is shown in closed circles and the Not-ASD group in open circles. Note that the ASD group showed a greater variation in the percentage of time spent in the periphery, accounting for the fact that it was the group that had the strongest effect. Note also that the dependent measures showed a ceiling effect past about 50% time spent in the periphery which would also influence the strength of the correlation.

**Figure 4:**
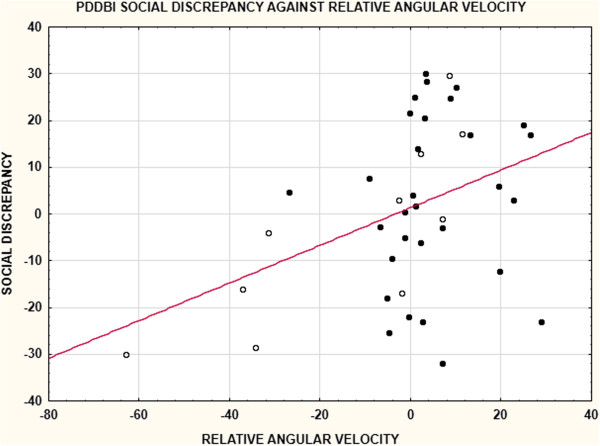
**This figure shows the relation between the PDDBI Social Discrepancy score and relative angular velocity.** ASD cases are in filled circles and Not-ASD cases in open circles. The regression function is for the entire sample. Note that relative angular velocity has more variation in the Not-ASD group than in the ASD group accounting for the stronger correlation for the Not-ASD group in Table 5.

Surprisingly, RAV was positively correlated with measures of maladaptive social communication but this effect depended on the measure, group, and time period as shown in Table 5. For the parent ratings, the effects were seen only for RAV1 and, for the PDDBI, effects were present only in the Not-ASD group. For the ADOS-G, the effects were seen only for the second 3-min phase after the ADOS-G session was over and only for the group as a whole. This positive correlation indicated that the greater the angular velocity towards the left, the worse the maladaptive social behavior scores. As shown in Figure 4, this complicated relationship for the PDDBI and the major effect for the Not-ASD group overall was due to range restriction for RAV in the ASD group.

### Tracking data and adaptive social communication

For adaptive skills (Table 6), the percentage of time spent in the periphery was inversely correlated with social, self-care, and language skills across the PDDBI and VABS-II with the effect driven by the ASD group. Latency to approach the parent was inversely correlated with the VABS-II domains while latency to approach the periphery was positively associated with these adaptive skills, again only in the ASD group. The more time children spent in the periphery, the worse their scores, while the longer it took for them to enter the periphery, the better their scores. There was a weak, but significant negative correlation between overall RAV and Social Approach Behaviors as measured by the PDDBI.

**Table 6:** Correlations between tracking measures and adaptive social communication skills for all data: All (n = 40), ASD (n = 30, and Not-ASD (n = 10) subsets

**Adaptive**	**Parent**		**Periphery**		**Turning bias**		
	**LAT**	**DUR%**	**LAT**	**DUR%**	**RAV1**	**RAV2**	**RAV MEAN**
PDDBI Social Approach Behaviors							
All	−0.18	−0.11	0.21	−0.38	−0.28	−0.16	**−**0.31
*P*	0.276	0.492	0.193	0.014	0.079	0.327	0.054
ASD	−0.00	−0.24	0.27	−0.43	−0.05	0.12	0.06
*P*	0.993	0.198	0.149	0.018	0.785	0.528	0.750
Not-ASD	−0.33	0.08	−0.13	0.46	−0.57	0.05	−0.47
*P*	0.355	0.821	0.730	0.183	0.088	0.892	0.172
PDDBI Expressive Language							
All	−0.28	0.04	0.40	−0.59	−0.24	−0.10	−0.24
*P*	0.080	0.808	0.011	0.000	0.128	0.582	0.143
ASD	−0.18	0.04	0.48	−0.64	−0.20	0.27	0.08
*P*	0.334	0.834	0.007	0.000	0.299	0.156	0.686
Not-ASD	0.17	−0.34	−0.09	0.54	−0.38	0.19	−0.25
*P*	0.640	0.332	0.807	0.106	0.278	0.600	0.491
PDDBI Learning, Memory, and Receptive Language							
All	−0.12	−0.15	0.19	−0.45	−0.27	0.02	−0.18
*P*	0.479	0.368	0.234	0.004	0.089	0.899	0.255
ASD	0.01	−0.29	0.22	−0.43	−0.29	0.28	0.02
*P*	0.967	0.120	0.248	0.017	0.125	0.135	0.906
Not-ASD	−0.11	0.42	−0.15	0.11	−0.34	0.38	−0.13
*P*	0.767	0.230	0.689	0.756	0.343	0.274	0.731
VABS-II Communication							
All	−0.41	0.15	0.45	−0.69	−0.28	−0.08	−0.26
*P*	0.008	0.371	0.004	0.000	0.078	0.634	0.112
ASD	−0.36	0.19	0.49	−0.74	−0.17	0.25	0.08
*P*	0.050	0.312	0.006	0.000	0.362	0.185	0.677
Not-ASD	−0.04	−0.32	0.29	0.26	−0.66	−0.19	−0.65
*P*	0.921	0.363	0.418	0.475	0.040	0.591	0.042
VABS-II Daily Living Skills							
All	−0.44	0.22	0.31	−0.65	−0.19	−0.04	−0.17
*P*	0.004	0.165	0.051	0.000	0.232	0.793	0.300
ASD	−0.41	0.26	0.31	−0.63	−0.12	0.17	0.05
*P*	0.026	0.161	0.096	0.000	0.519	0.372	0.793
Not-ASD	−0.04	−0.17	0.41	−0.39	−0.44	0.30	−0.25
*P*	0.922	0.643	0.246	0.271	0.205	0.403	0.485
VABS-II Socialization							
All	−0.47	0.13	0.34	−0.63	−0.16	−0.14	−0.21
*P*	0.002	0.411	0.031	0.000	0.310	0.384	0.191
ASD	−0.40	0.12	0.41	−0.65	−0.14	0.17	0.04
*P*	0.030	0.536	0.023	0.000	0.447	0.365	0.848
Not-ASD	−0.39	0.04	−0.32	0.19	−0.09	−0.07	−0.11
*P*	0.266	0.905	0.374	0.606	0.798	0.851	0.763

LAT = Latency (sec); DUR% = Percentage of session time; RAV = Relative angular velocity (°/sec).

### Tracking data and repetitive and ritualistic behaviors

Correlations between the tracking data and measures of stereotyped and ritualistic behaviors are shown in Table 7. As above, the tracking measure showing the greatest generality across scales was the percentage of time spent in the periphery and it was linked more to sensory than to ritualistic type behaviors, i.e., behaviors likely associated with problems with the arousal system rather than with anxiety [17] and the effect was most evident in the ASD group. Although RAV was meant to pick up this behavior, and the correlations were in the expected direction, they were weak and did not reach statistical significance in the group as a whole. In the Not-ASD group, however, RAV was positively correlated with parent, but not with ADOS, ratings. Thus, the more the Not-ASD children were reported to exhibit repetitive behaviors, the more they showed a left-turn bias, similar to the ASD group. As with the effects of RAV on Maladaptive Social Communication, this group difference was due to range restriction in the ASD group.

**Table 7:** Correlations between tracking measures and repetitive and ritualistic behaviors severity for all data: All (n = 40), ASD (n = 30), and Not-ASD (n = 10) subsets

	**Parent**		**Periphery**		**Turning bias**		
s	**LAT**	**DUR%**	**LAT**	**DUR%**	**RAV1**	**RAV2**	**RAV MEAN**
PDDBI Sensory/Perceptual Approach Behaviors							
All	0.16	−0.09	−0.25	0.47	0.21	0.14	0.25
*P*	0.312	0.585	0.119	0.002	0.185	0.390	0.126
ASD	0.18	−0.19	−0.18	0.52	0.04	0.03	0.05
*P*	0.331	0.309	0.338	0.003	0.842	0.868	0.779
Not-ASD	−0.00	0.28	−0.63	0.19	0.50	0.43	0.62
*P*	0.998	0.430	0.051	0.595	0.143	0.213	0.058
PDDBI Ritualisms/Resistance to Change							
All	−0.02	−0.01	−0.22	0.14	0.18	−0.02	0.12
*P*	0.925	0.973	0.168	0.373	0.259	0.811	0.472
ASD	0.16	−0.19	0.22	0.33	−0.06	0.17	0.10
*P*	0.413	0.326	0.251	0.079	0.761	0.356	0.594
Not-ASD	−0.02	0.30	−0.51	0.09	0.63	0.35	0.69
*P*	0.965	0.401	0.136	0.800	0.053	0.319	0.026
ABC/C Stereotypy							
All	0.21	−0.09	−0.43	0.62	0.31	−0.04	0.20
*P*	0.184	0.586	0.005	0.000	0.055	0.808	0.225
ASD	0.31	−0.17	−0.39	0.69	0.23	−0.09	0.09
*P*	0.101	0.375	0.033	0.000	0.214	0.629	0.625
Not-ASD	−0.18	0.18	−0.73	0.47	0.55	0.19	0.56
*P*	0.629	0.610	0.016	0.174	0.103	0.591	0.096
ADOS-G Restricted and Repetitive Behaviors							
All	0.14	−0.00	−0.16	0.40	0.17	−0.00	0.12
*P*	0.383	0.976	0.316	0.011	0.288	0.986	0.449
ASD	0.04	−0.03	−0.16	0.36	0.13	−0.32	−0.17
*P*	0.852	0.861	0.385	0.049	0.487	0.086	0.381
Not-ASD	−0.46	0.56	−0.10	−0.06	0.14	−0.64	−0.16
*P*	0.186	0.090	0.784	0.872	0.710	0.046	0.661

LAT = Latency (sec); DUR% = Percentage of session time; RAV = Relative angular velocity (°/sec).

### Tracking data and autism severity

**Figure 5:**
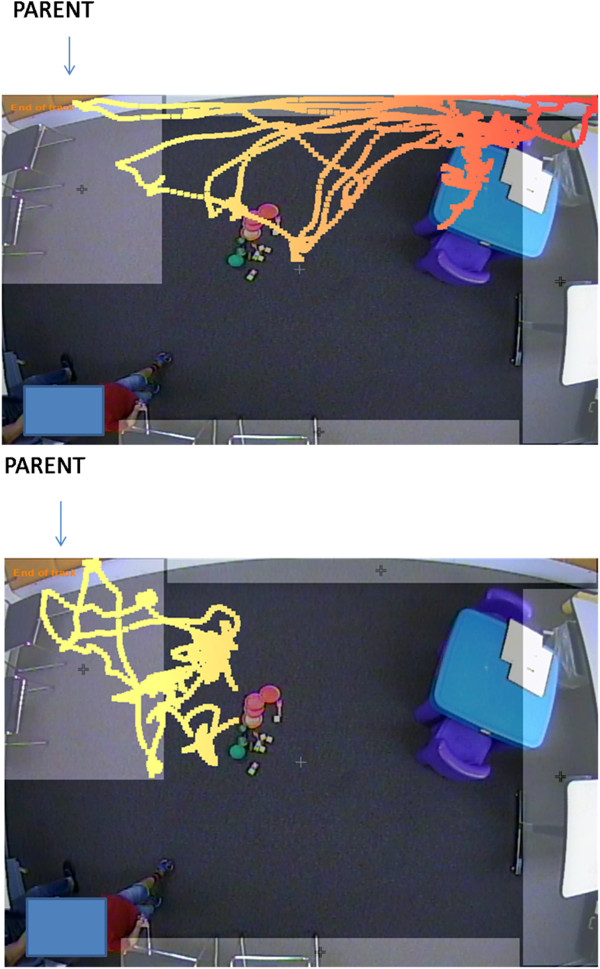
**Paths taken in the first of two 3-min intervals prior to ADOS-G assessment in two 2-year-old boys.** Color indicates distance from parent ROI (yellow is closer). Child on top has ASD, child on bottom has a language delay.

There were two measures related to severity of autism: the Autism composite score on the PDDBI and the Comparison Score on the ADOS-G (the higher the scores, the greater the severity). Their associations with the tracking data are shown in Table 8. Only one of the social measures, latency to approach the parent, was associated with the ADOS-G measure (the greater the latency the worse the Comparison Score) and this effect was driven by the ASD group. The percentage of time spent in the periphery was again broadly linked to autism severity across the PDDBI and ADOS-G scales but especially evident in the ASD group. The more time children spent in the periphery, the worse their autism severity scores.Examples of this relation between periphery preference and diagnosis are shown in Figure 5, which shows, the various locations of two different boys, both 2 years of age, during the first 3 min. The color of their paths indicates distance from the parent ROI with yellow closer than orange. The child on the bottom is moving between the toys on the floor and his parent while the child on the top is moving toys from the table to the floor and back again in a repetitive pattern, all while remaining close to the one-way mirror. The child on the top was diagnosed with ASD and spent 18% of this period in the periphery while the one on the bottom had a language delay and spent no time in the periphery.

**Table 8:** Correlations between tracking measures and autism severity for all data: All (n = 40), ASD (n = 30), and Not-ASD (n = 10) subsets

	**Parent**		**Periphery**		**Turning bias**		
	**LAT**	**DUR%**	**LAT**	**DUR%**	**RAV1**	**RAV2**	**RAV MEAN**
PDDBI Autism							
All	0.20	0.04	−0.23	0.30	0.36	0.19	0.39
*P*	0.217	0.801	0.146	0.058	0.022	0.228	0.013
ASD	0.24	−0.07	−0.21	0.45	0.02	0.15	0.14
*P*	0.204	0.729	0.277	0.013	0.899	0.426	0.454
Not-ASD	0.20	0.27	−0.41	−0.12	0.70	0.43	0.79
*P*	0.581	0.447	0.239	0.743	0.024	0.219	0.007
ADOS-G Comparison Score							
All	0.41	−0.11	0.09	0.35	0.11	0.48	0.39
*P*	0.009	0.517	0.591	0.026	0.516	0.002	0.014
ASD	0.36	−0.11	0.27	0.28	−0.02	0.16	0.12
*P*	0.053	0.574	0.151	0.132	0.917	0.395	0.536
Not-ASD	−0.33	0.26	0.01	0.33	−0.31	−0.44	−0.46
*P*	0.351	0.463	0.979	0.355	0.381	0.207	0.184

LAT = Latency (sec); DUR% = Percentage of session time; RAV = Relative angular velocity (°/sec).

RAV was also linked with autism severity across these two instruments suggesting that turning bias is related to the social deficits seen in autism since this behavior was linked to both autism severity and to social communication problems. The magnitude of this effect depended on the observation interval with the correlation strongest for the PDDBI during the first 3 min (driven by the Not-ASD group) while it was strongest for the ADOS-G rating during the second 3 min phase (for the entire sample). The greater the angular velocity towards the left, the worse the autism severity scores across the entire sample. Again, ASD range restriction for RAV played a role similar to the effects noted above.

### Tracking data and not-ASD specific behavior problems

The PDDBI and the ABC-C provide additional information on behavior problems not uniquely related to ASD. These include problems with arousal regulation, fears, and aggressivity; Table 9 shows these correlations. Problems with arousal regulation (hyperactivity, sleeping problems, etc.) were associated primarily with measures related to remaining in the periphery (both latency and percent time) and not to the parent measures with effects driven by the ASD group. The more time children spent in the periphery, the worse their arousal scores. There was no significant relation with overall RAV but there were small positive correlations between measures of arousal regulation and RAV in the first 3 min such that the greater the angular velocity towards the left, the worse the severity of ratings of arousal problems, with the effects driven by the Not-ASD group (again due to range restriction for RAV in the ASD group as discussed above). Ratings of fears were not associated with the tracking data. Aggressivity was correlated with increased time spent in the periphery only for the ASD group.

**Table 9:** Correlations between tracking measures and Not-ASD specific severity of behavior problems for all data: All (n = 40), ASD (n = 30), and Not-ASD (n = 10) subsets

	**Parent**		**Periphery**		**Turning bias**		
**AROUSAL**							
	**LAT**	**DUR%**	**LAT**	**DUR%**	**RAV1**	**RAV2**	**RAV MEAN**
PDDBI Arousal Regulation Problems							
All	0.01	0.02	−0.41	0.35	0.17	−0.15	0.03
*P*	0.933	0.926	0.009	0.026	0.286	0.350	0.867
ASD	0.09	0.02	−0.39	0.42	−0.02	−0.05	−0.06
*P*	0.651	0.928	0.034	0.020	0.898	0.809	0.771
Not-ASD	−0.11	−0.08	−0.67	0.27	0.79	−0.25	0.57
*P*	0.762	0.832	0.035	0.450	0.007	0.48	0.083
ABC/C Hyperactivity							
All	0.19	−0.16	−0.35	0.45	0.32	−0.21	0.09
*P*	0.237	0.335	0.027	0.004	0.044	0.185	0.564
ASD	0.38	−0.31	−0.36	0.63	0.31	−0.09	0.15
*P*	0.039	0.090	0.051	0.000	0.098	0.638	0.434
Not-ASD	0.17	0.13	−0.60	0.51	0.63	0.12	0.59
*P*	0.632	0.723	0.065	0.130	0.053	0.742	0.071
FEARS							
PDDBI Specific Fears							
All	−0.16	0.10	−0.20	0.07	0.20	−0.23	−0.00
*P*	0.316	0.548	0.213	0.672	0.210	0.158	0.999
ASD	−0.06	−0.07	−0.23	0.23	0.06	0.05	0.09
*P*	0.740	0.704	0.226	0.214	0.749	0.781	0.646
Not-ASD	−0.01	0.39	−0.36	0.05	0.54	−0.19	0.39
*P*	0.973	0.260	0.314	0.895	0.104	0.606	0.265
AGGRESSION							
PDDBI Aggressiveness							
All	−0.08	0.14	−0.11	0.22	0.09	−0.26	−0.10
*P*	0.636	0.373	0.505	0.168	0.586	0.106	0.525
ASD	0.12	−0.02	−0.15	0.48	0.13	−0.12	−0.00
*P*	0.499	0.927	0.444	0.007	0.487	0.535	0.994
Not-ASD	−0.32	0.46	−0.13	−0.20	0.22	−0.02	0.18
*P*	0.362	0.181	0.713	0.587	0.550	0.963	0.621
ABC/C Irritability							
All	0.07	−0.01	−0.16	0.16	0.28	−0.13	0.12
*P*	0.662	0.964	0.310	0.318	0.079	0.416	0.468
ASD	0.32	−0.25	−0.16	0.41	0.24	0.09	0.25
*P*	0.081	0.180	0.398	0.026	0.193	0.637	0.183
Not-ASD	0.12	0.33	−0.46	0.18	0.57	0.27	0.61
*P*	0.749	0.354	0.179	0.620	0.082	0.445	0.059

LAT = Latency (sec); DUR% = Percentage of session time; RAV = Relative angular velocity (°/sec).

## Discussion

These results suggest that data obtained from automated tracking of motion of children on the autism spectrum can serve as a valid indicator of the severity of their disorder as well as their problems with arousal regulation and irritability. They also suggest that time spent in the periphery is associated with ASD severity.

Indeed, the average percentage of time spent in the periphery of the room was the one measure that showed cross-scale consistency for a variety of both maladaptive and adaptive behaviors in the expected direction. As this percentage increased, ratings of the severity of stereotypies, social avoidance and social interaction problems, autism severity, and hyperactivity and general arousal regulation problems moderately increased while ratings of social and linguistic competence strongly decreased. By contrast, the percentage of time spent in the vicinity of the parent was of limited value and showed no relation with any of the measures. Latency to approach the parent was, however, linked to overall adaptive social communication skills as well as to the ADOS-G Social Affect and Comparison scores indicating that those with better communication skills were more likely to quickly approach or be near their parent once the examiner left the room.Thus, both latency to approach the parent and the percentage of time the child spends in the periphery of a room may serve as objective indicators of autism severity, important measures to study as possible predictors of the development of ASD in at-risk children, and as indicators of the effects of intervention. Ideally, intervention would shorten the parent latency as well as the time spent in the periphery (e.g., making the path of the ASD child look like the path of the Not-ASD child in Figure 5). Both latency and duration measures were invariant across the pre- and post-ADOS assessment time periods.

In animal studies, the tendency to prefer the periphery of an open field is referred to as thigmotaxis and usually serves as an indicator of anxiety [21]. In this study, there was no significant correlation between parent reports of fears on the PDDBI and percentage of time spent in the periphery. Instead, there was an association between percentage of time spent in the periphery and stereotyped behaviors, as well as with measures of arousal regulation and irritability. It may be that children with ASD, when introduced into a novel room, spend the time exploring the periphery because that is where the interesting sensory stimuli are; in our case, the one-way mirror, storage cabinets, covered toys, and walls. It could also be that engaging in such behavior serves as a means of regulation of their arousal and/or anxiety which is known to be elevated in children on the autism spectrum [22-24].

RAV was the one measure that differed across time periods with a marked increase in velocity in the second observation period relative to the first and moving from a neutral bias to a left-sided bias but only in the ASD group. It is unclear why the overall increase in turning rate occurred but could be related to an “overflow” of arousal generated by the social demands of ADOS testing for the ASD group. This may account for the observation that the clinician’s ratings of social affect on the ADOS were correlated more with the second time-period than the first. Indeed, RAV after the ADOS was strongly associated with diagnosis in this sample.

Based on the work of Bracha et al. [1], we had expected RAV to be linked with ratings of stereotyped behaviors in the ASD group. These authors reported that spinning in children with ASD had a left turn bias which they attributed to right sided neglect. However, we did not see a link with ratings of stereotyped behaviors in the ASD group but this was largely because they showed relatively little variation in RAV. Instead, RAV was associated with parental reports of stereotyped behaviors (and to social deficits) in the Not-ASD group where there was much greater variation in RAV across subjects.

Finally, we acknowledge that the numbers of correlations within each table were quite large. As noted above, correction for multiple comparisons would have been too strict an approach for this exploratory study. A more informal approach toward handling this issue is to compare the expected number of significant effects for a *P* value of 0.05 relative to that obtained, as suggested by Gelman, Hill, and Yajima [25]. Tables 5 and 6 each had 126 correlations and so we would expect each to have six significant correlations by chance. Instead, Table 5 had 25 significant correlations and Table 6 had 28. Table 7 would be expected to have four significant correlations by chance but 12 were significant. The expected chance significance rate was two for Table 8 and five for Table 9, but the actual numbers were 10 and 15, respectively. Based on these observations and the observed generalization across scales, we conclude that our results are likely to be valid.

## Conclusions

Automated detection of very basic measures of behavior, such as latency to approach a caregiver, time spent in the periphery of a room with the parent present, and, perhaps, speed of turning toward the left, may serve as valid markers of the severity of social, repetitive, and arousal-based problem behaviors in children with ASD. Since these measures can be readily computed and do not involve specific tasks, they may serve as unbiased, culture-free indicators of early signs of ASD or as indicators of change with intervention.

The number of tracking measures we selected was arbitrarily limited due to our relatively small sample size, focusing on ones we thought would be most relevant. Other measures that may also be of value include measures of path complexity, relative frequency of slow and fast movements, distance from the parent or periphery, and concurrent assessment of psychophysiological measures of arousal, amongst others. The latter would be of help in ascertaining the extent to which arousal regulation helps to explain our findings.

We do not know to what extent our results are impacted by the size or layout of the observation room or the shapes and sizes of the ROIs. More research is needed to investigate such effects as well as the need to replicate our observations, to examine associations of our measures with social bids and repetitive behaviors exhibited during the observation periods, to examine the contributions of our measures to diagnosis, and to assess their sensitivity to intervention effects.

## Abbreviations

ABC/C: Aberrant Behavior Checklist-Community; ADOS-G: Autism Diagnostic Observation Schedule-Generic; ASD: Autism Spectrum disorder; PDDBI: PDD Behavior Inventory; RAV: Relative angular velocity; ROI: Region of interest; VABS-II: Vineland Adaptive Behavior Scales, Second Edition.

## Competing interests

The PDDBI generates a royalty for Dr. Cohen. Drs. Gardner, Karmel, and Kim report no financial interests or potential competing interests.

## Authors’ contributions

ILC first floated the idea of using automated detection to examine preference for the periphery in cases with ASD, examined the participants, gathered the data, helped to evaluate and set up the Noldus Ethovision XT system, set up the arena partitions, chose the variables for analysis, performed the data analyses, and wrote the manuscript. JMG helped to develop the observation protocol. BZK also helped to develop the observation protocol. SYK helped with selection of the variables of interest, generating software for computing some of these variables. All authors read and approved the final manuscript.

## Authors’ information

ILC is a behavioral psychologist with training in neuroscience, conditioning and learning, and clinical psychology. He has extensive experience in the study of ASD. JMG is a developmental psychologist who has a long history of studying arousal and perception in infancy and who currently studies infants at-risk for developmental problems. BZK is a developmental psychophysiologist with an extensive history of developing new measures to detect infant movement and perception and in detecting developmental anomalies in infants at-risk. SYK is a cognitive psychologist with a strong interest in developmental disabilities.
